# Optimized plasma preparation is essential to monitor platelet-stored molecules in humans

**DOI:** 10.1371/journal.pone.0188921

**Published:** 2017-12-08

**Authors:** Marion Mussbacher, Waltraud C. Schrottmaier, Manuel Salzmann, Christine Brostjan, Johannes A. Schmid, Patrick Starlinger, Alice Assinger

**Affiliations:** 1 Department of Vascular Biology and Thrombosis Research, Center of Physiology and Pharmacology, Medical University Vienna, Vienna, Austria; 2 Department of Surgery, General Hospital, Medical University Vienna, Vienna, Austria; Queen Mary University of London, UNITED KINGDOM

## Abstract

Platelets store a plethora of different molecules within their granules, modulating numerous pathways, not only in coagulation, but also in angiogenesis, wound healing, and inflammatory diseases. These molecules get rapidly released upon activation and therefore represent an easily accessible indirect marker for platelet activation. Accurate analysis of platelet-derived molecules in the plasma requires appropriate anticoagulation to avoid *in vitro* activation and subsequent degranulation of platelets, potentially causing artificially high levels and masking biologically relevant differences within translational research studies. However, there is still enormous heterogeneity among anticoagulants used to prevent unwanted platelet activation, so that plasma levels reported for platelet granule contents range over several orders of magnitude. To address this problem and to define the most robust method of plasma preparation to avoid *in vitro* platelet activation during processing, we compared plasma concentrations of the three platelet-stored factors thrombospondin (TSP-1), platelet factor 4 (PF4), and soluble P-selectin (sCD62P) between human blood samples anticoagulated with either citrate-theophylline-adenosine-dipyridamole (CTAD), acid-citrate-dextrose (ACD), citrate, ethylenediaminetetraacetic acid (EDTA) or heparin. Additionally, we assessed the effect of storage temperature and time between blood drawing and sample processing within the differentially anticoagulated samples. Our data strongly support the use of CTAD as anticoagulant for determining plasma concentrations of platelet-stored molecules, as anticoagulation with heparin or EDTA led to a 12.4- or 8.3-fold increase in plasma levels of PF4, respectively. Whereas ACD was similar effective as CTAD, citrate only showed comparable PF4 plasma levels when plasma was kept at 4°C. Moreover, blood sampling with CTAD as anticoagulant resulted in the most reproducible values, even when samples were processed at ambient temperature or after storage over 6 hours. In the latter case, anticoagulation with heparin or EDTA led to artificially high plasma levels indicative of *in vitro* platelet activation. Therefore, we want to raise scientific awareness for choosing CTAD as optimal anticoagulant for the detection of platelet-stored molecules in plasma.

## Introduction

Precise measurement of plasma parameters is not only important for accurate diagnosis, but also provides a non-invasive, easily applicable tool to monitor disease progression and to possibly predict clinical outcome [1]. Moreover, most clinical studies include plasma parameters in their study design and billions of plasma samples are stored within biobanks for further analysis [2, 3].

During the past few years, platelets and microvesicles have been identified as an important source of mediators, modulating numerous pathways, not only in coagulation, but also in angiogenesis, wound healing, and immune diseases [4–8]. Upon platelet activation, a plethora of molecules is released from intracellular granules, including TSP-1, PF4, vascular endothelial growth factor (VEGF), serotonin, and CD62P [9]. As platelet granules store over 300 different molecules, degranulation has to be a precisely coordinated process, adjusted to the specific physiological needs [10, 11]. This is not only assured by distinct granule types (α-granule, dense granule, and lysosomes), but also *via* diverse packaging and agonist-dependent release of granule subtypes [7]. Moreover, since platelets store molecules with opposing functions (e.g. pro- and antiangiogenic; pro-and anti-inflammatory) time-dependent platelet secretion profiles have been considered important for clinical outcome [12]. However, due to the high sensitivity of platelets and their quick adaption to changes in the microenvironment, they are prone to *in vitro* activation, which has to be considered when preparing plasma for analysis of platelet-derived mediators. Therefore, prevention of platelet activation and complete removal of platelets is equally important for plasma preparation as preserving protein stability and enzyme activity of plasma components.

While this has been known for decades, there is still enormous discrepancy between published plasma levels of platelet-stored molecules, ranging over several orders of magnitude [13]. This broad variation makes it difficult to compare different studies and leads to highly controversial findings.

Therefore, optimized plasma preparation is crucial to measure differences in platelet granule release and to avoid artifacts due to *in vitro* platelet activation, which would mask biological differences that occurred *in vivo*. Moreover, appropriate plasma preparation helps to reduce sample variations and increases the reproducibility of results.

To address this issue, we compared different plasma preparation protocols with special emphasis on appropriate anticoagulants as well as storage temperature to avoid *in vitro* platelet activation, additionally considering time between blood drawing and sample processing.

## Methods

### Study collective of healthy volunteers

All healthy volunteers (5 male, 3 female donors aged between 26–51 years) were free of any medication for at least 2 weeks and gave their informed consent. Venous blood was collected from the antecubital vein using a 24G needle and the indicated tubes for anticoagulation. The study was approved by the Human Ethics Committee of the Medical University of Vienna (EK237/2004) and complied with the Declaration of Helsinki.

### Plasma preparation

**Fig 1:**
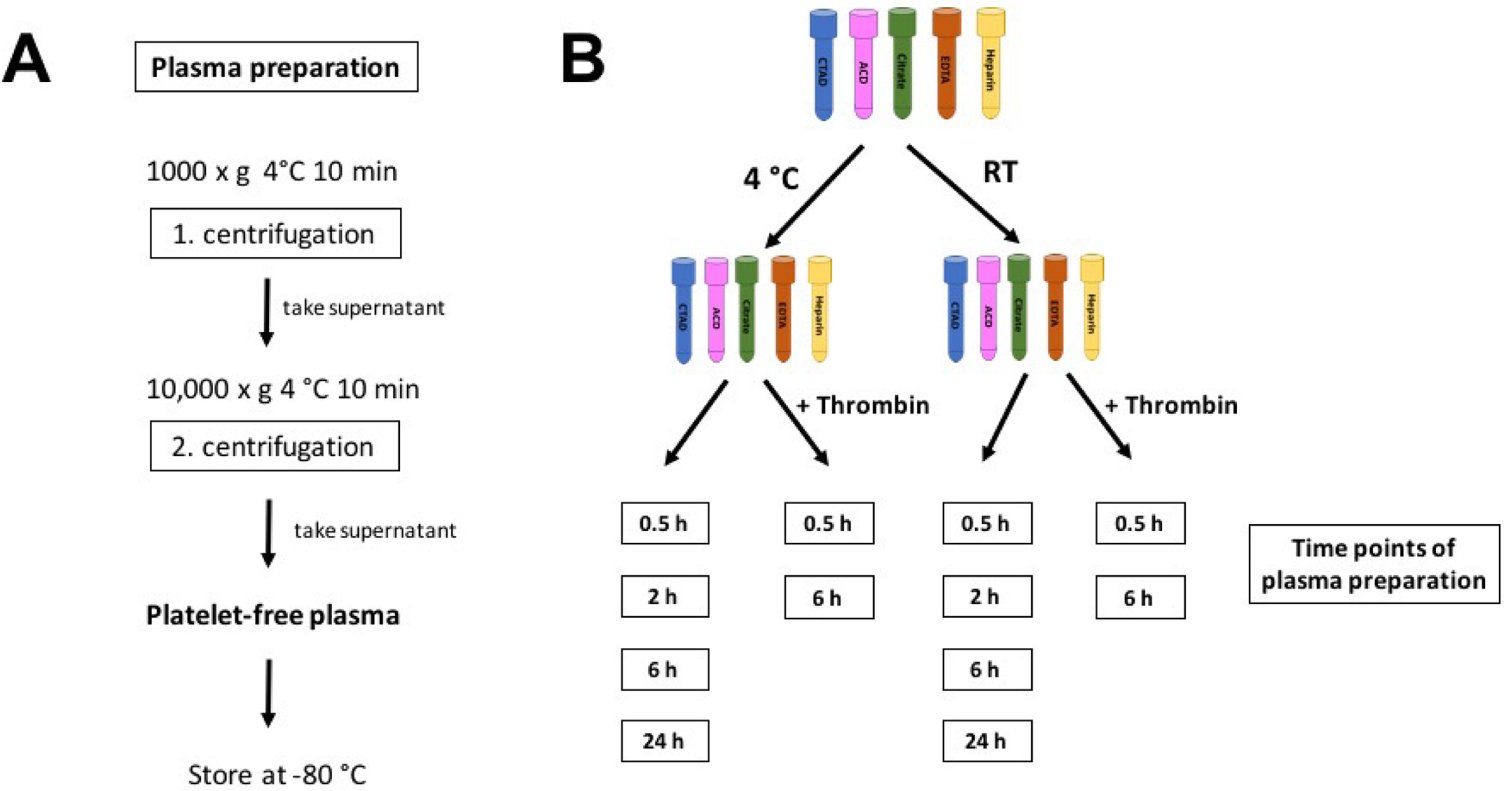
Experimental set-up to compare the effect of different anticoagulants on plasma levels of platelet-stored factors. (A) Plasma preparation. Anticoagulated blood of 8 healthy volunteers was centrifuged at 1,000 x g and 4°C for 10 minutes. Supernatants were transferred to new tubes and centrifuged at 10,000 x g and 4°C for 10 minutes to generate platelet-free plasma. Supernatants of the second centrifugation step were aliquoted and stored at –80°C until further use. (B) Sampling. Blood of each donor was collected using CTAD (blue), ACD (pink), citrate (green), EDTA (red) and heparin (yellow) as anticoagulant. Half of the samples were kept either at 4°C or at room temperature. Plasma was generated at 0.5 h, 2 h, 6 h, and 24 h. To analyze the effect of *in vitro* platelet activation a subgroup of samples was stimulated with thrombin at a submaximal concentration and plasma was generated after 0.5 h and 6 h.

Depending on the experimental set-up for plasma preparation (see Fig 1), blood was drawn into pre-chilled or room temperature (RT) citrate-theophylline-adenosine-dipyridamole (CTAD), acid-citrate-dextrose (ACD), 3.8% citrate, dipotassium ethylenediaminetetraacetic acid (EDTA) or sodium heparin tubes. Blood samples were centrifuged at the indicated time-points at 1,000 x g and 4°C for 10 min. After this first centrifugation step, the plasma supernatant was transferred to a new vial and subjected to a second round of centrifugation at 10,000 x g and 4°C for 10 min to guarantee removal of all cellular components. Samples labeled as 4°C were always kept on ice, while RT indicates that samples were not cooled between centrifugation steps. The supernatant was stored in aliquots at -80°C until further use.

### *In vitro* activation of platelets

Within 30 minutes after blood drawing, platelets were stimulated in whole blood with a submaximal dose of thrombin (0.1 U/mL) to trigger platelet activation *in vitro*.

### Determination of plasma concentrations

Plasma concentrations of TSP-1, PF4, and sCD62P were analyzed with enzyme-linked immunosorbent assay (ELISA) using commercially available ELISA kits (Quantikine; R&D Systems, Minneapolis, MN, U.S.A.) according to the manufacturer´s instructions.

### Statistical analysis

Data are presented as medians with interquartile ranges and were analyzed with Graph Pad Prism 6 using one-way and two-way ANOVA with a Dunnet correction. * p values < 0.05 were considered as statistically significant.

## Results

### Plasma levels of platelet-stored factors are substantially increased after non-optimized plasma preparation and highly sensitive to temperature variations

**Fig 2:**
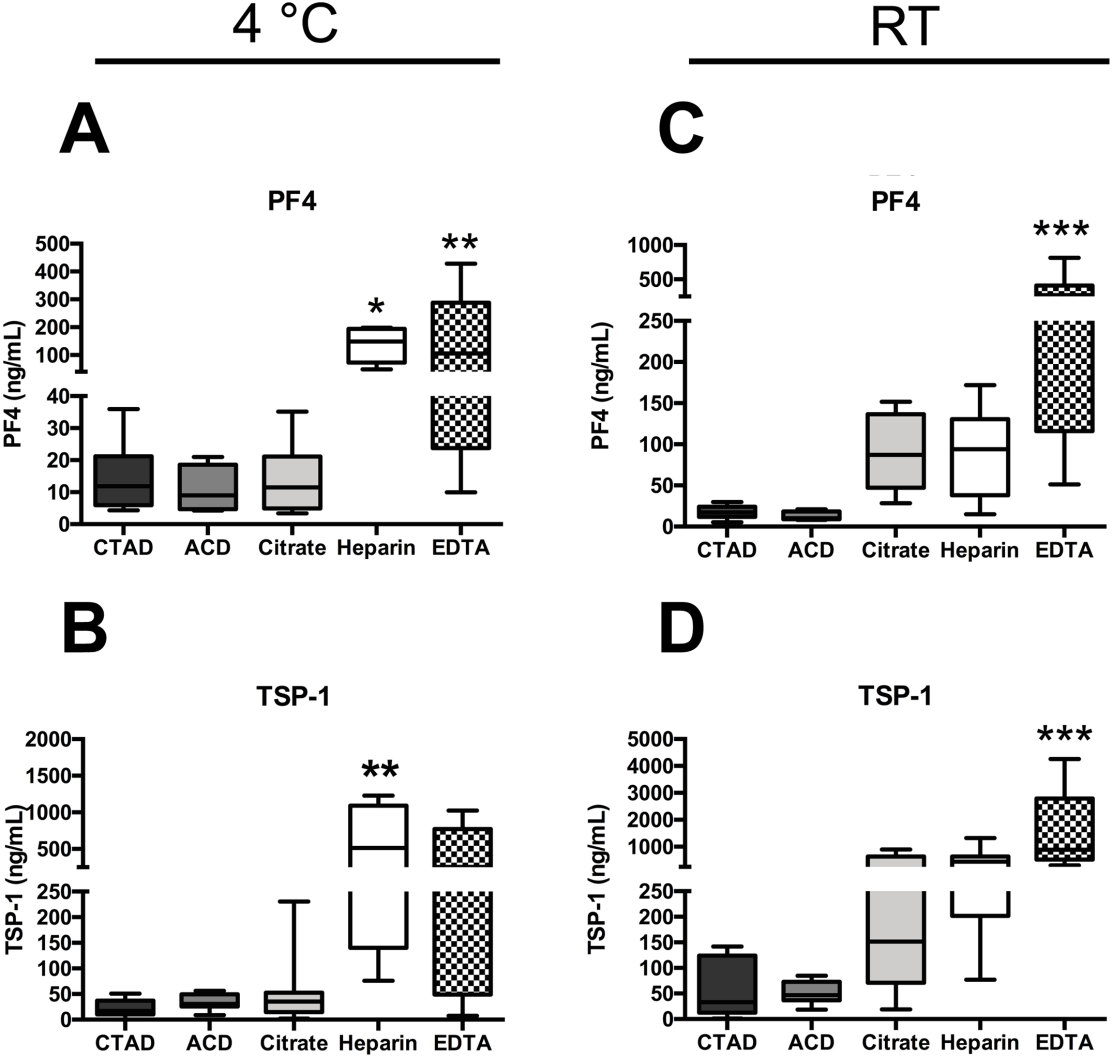
Plasma levels of platelet-stored factors vary among different anticoagulants. Plasma levels of PF4 (A, C) and TSP-1 (B, D) were determined in 8 healthy individuals. Plasma was prepared from CTAD (black bar), ACD (dark grey bar), citrate (grey bar), heparin (white bar) or EDTA (patterned bar) blood at 4°C (A-B) or at room temperature (C-D) within 30 min after blood draw. Plasma concentration was measured using ELISA. Significant differences were analyzed using one-way ANOVA with Dunnett correction and were depicted as *p<0.05, **p<0.01, ***p<0.001 (in comparison to CTAD).

To test for the effect of different anticoagulants on the level of platelet-stored molecules, we compared different anticoagulants in pre-chilled vials (Figs 1, 2A and 2B, Table 1).

**Table 1:** 

	PF4 (ng/mL)		TSP-1 (ng/mL)	
	median (range)	p-value	median (range)	p-value
**4°C**				
CTAD	12 (4–36)		25 (0–144)	
ACD	7 (4–10)	0.9999	31 (9–56)	0.9999
Citrate	12 (3–35)	0.9999	35 (2–234)	0.9982
Heparin	149 (48–198)	0.0090	514 (76–1228)	0.0013
EDTA	106 (10–428)	0.0032	254 (7–1025)	0.0484
**RT**				
CTAD	17 (5–30)		33 (1–142)	
ACD	10 (8–21)	0.9999	47 (18–85)	>0.9999
Citrate	87 (28–152)	0.5215	152 (19–899)	0.8733
Heparin	94 (15–172)	0.5309	444 (77–1326)	0.4996
EDTA	269 (51–814)	<0.0001	886 (306–4258)	0.0003

Plasma PF4 and TSP-1 levels showed a significant correlation with different anticoagulants (p = 0.0003 for PF4 and p = 0.0003 for TSP-1). Whereas plasma levels of PF4 and TSP-1 were comparable between CTAD, ACD and citrate plasma, these proteins substantially increased when heparin or EDTA were used as an anticoagulant (Fig 2A and 2B). Moreover, this was associated with an augmented inter-individual variation within the samples collected from EDTA plasma. Plasma levels of sCD62P were comparable between all four different groups (S1 Fig). When we further assessed platelet-stored molecules in different anticoagulants after processing at RT to evaluate the impact of temperature on *in vitro* platelet activation, the effect was even more pronounced (p<0.0001 for PF4; Fig 2C). In particular, we found elevated levels of PF4 and TSP-1 in non-CTAD preparations such as citrate, heparin, and EDTA plasma compared to plasma processed at 4°C (Fig 2C and 2D). In citrate plasma, PF4 and TSP-1 levels were increased 5.1-fold and 4.6-fold, respectively. Importantly, CTAD and ACD were the only anticoagulants that were not affected by changing processing temperature from 4°C to room temperature, as evidenced by a similar level of PF4 and TSP-1 under both conditions. No substantial fluctuations were observed for sCD62P concentrations (S1 Fig).

### Storage time-dependent differences in plasma levels of platelet-stored factors due to anticoagulants

**Fig 3:**
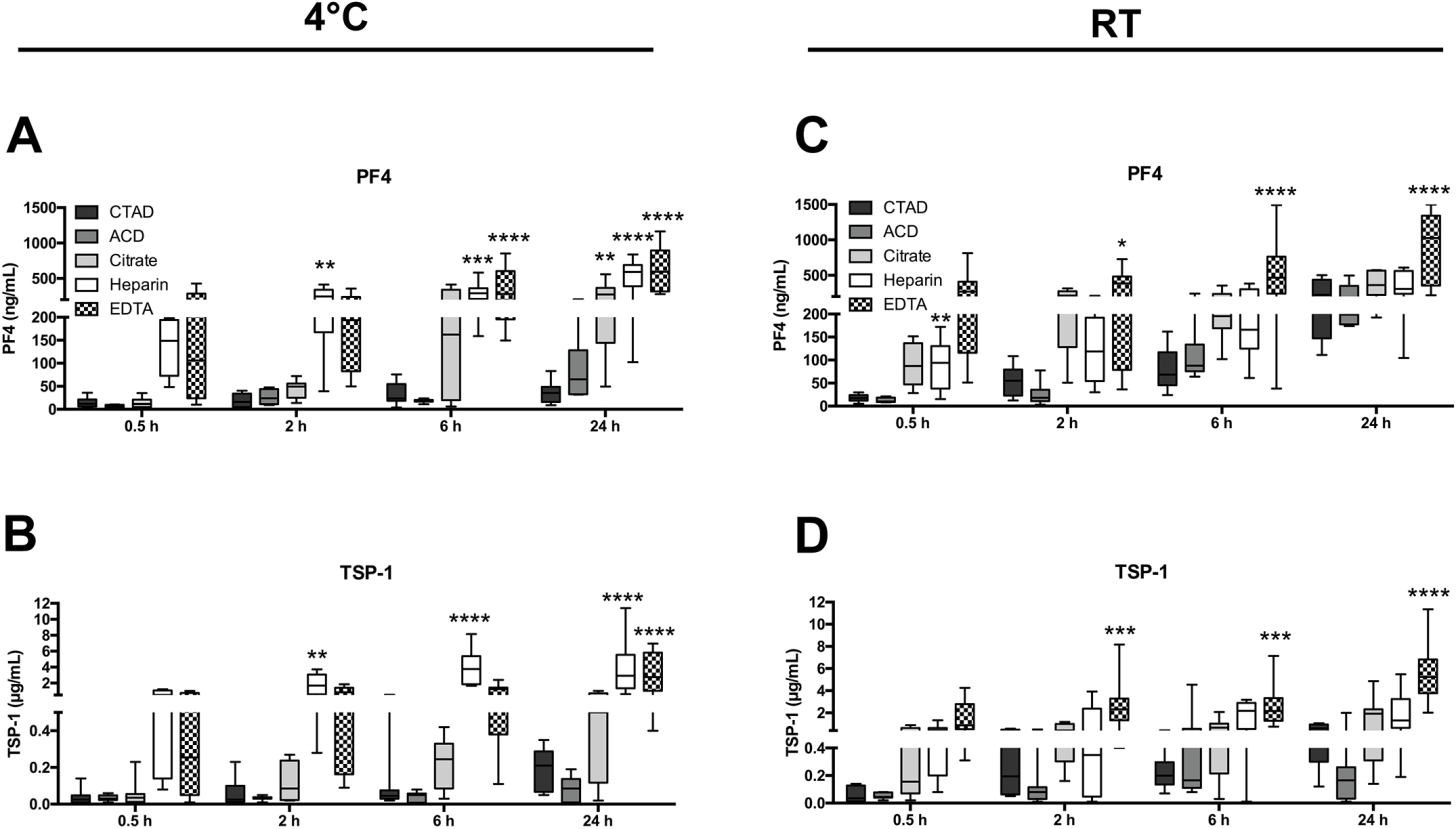
Time-dependent differences in plasma levels of platelet-stored factors. Blood from 8 healthy donors was anticoagulated with CTAD (black bar), ACD (dark grey bar), citrate (grey bar), heparin (white bar) or EDTA (patterned bar) and stored at 4°C (A-B) or at room temperature (C-D) for 0.5 h, 2 h, 6 h or 24 h until plasma preparation. Concentrations of PF4 (A, C) and TSP-1 (B, D) were determined for each time point. Significant differences were analyzed using two-way ANOVA with Dunnett correction (with CTAD as reference) and were depicted as *p<0.05, **p<0.01, ***p<0.001, and ****p<0.0001.

As plasma preparation is not always feasible immediately after blood drawing under clinical conditions, we tested the effect of storage on the release of platelet contents either at 4°C or at room temperature using different anticoagulants (Fig 3, Table 2). Both time and anticoagulant had a highly significant effect on the plasma levels of PF4 and TSP-1 (p<0.0001 for time and anticoagulants). Even though heparin and EDTA plasma showed already high levels of PF4 and TSP-1 under optimal timing conditions (within 30 minutes after blood drawing—see above), storage times between 2 hours and 24 hours resulted in a continuous increase in plasma levels, peaking at a concentration of 592 ng/mL and 3765 ng/mL as well as 595 ng/mL and 2752 ng/mL for PF4 and TSP-1, respectively (Fig 3A and 3B). In contrast, CTAD and ACD plasma levels of PF4 as well as TSP-1 remained constantly low even over a time period of 6 hours and only showed a moderate increase after 24 hours. Consistent with this finding, CTAD and ACD plasma exerted the smallest variation between different blood donors. At 4°C, we could neither observe any time-depended effects on plasma levels of sCD62P nor due to usage of certain anticoagulants (S2 Fig). Furthermore, we monitored time-dependent changes due to storage at room temperature and found a comparable increase in plasma levels of PF4 and TSP-1 in citrate, heparin, and EDTA plasma pointing to maximal granule release even under conditions of optimal cooling, which could not further be augmented due to storage at room temperature (Fig 3C and 3D). The only exceptions were CTAD and ACD plasma, in which we could only observe a moderate increase in PF4 levels over a time period of 6 hours (median 69 ng/mL and 88 ng/mL). However, CTAD was not able to prevent granule release after 24 hours at room temperature, pointing to the absolute limits of anticoagulation (median 225 ng/mL). In contrast to the other anticoagulants, plasma levels of TSP-1 remained relatively low in CTAD and ACD plasma over the whole experimental period.

**Table 2:** 

	PF4 (ng/mL)		TSP-1 (ng/mL)	
	median (range)	p-value	median (range)	p-value
**0.5 h, 4°C**				
CTAD	12 (3–36)		25 (0–144)	
ACD	7 (4–10)	0.9998	31 (9–56)	>0.9999
Citrate	12 (4–35)	>0.9999	35 (2–231)	>0.9999
Heparin	247 (39–198)	0.2001	514 (76–1228)	0.7431
EDTA	106 (10–428)	0.1279	254 (7–1025)	0.9315
**0.5 h, RT**				
CTAD	17 (5–30)		33 (1–142)	
ACD	10 (8–21)	>0.9999	47 (18–85)	>0.9999
Citrate	87 (28–152)	0.8509	152 (19–899)	0.9853
Heparin	94 (15–172)	0.8558	444 (77–1326)	0.8941
EDTA	269 (51–814)	0.0077	886 (306–4258)	0.0632
**2 h, 4°C**				
CTAD	16 (3–41)		24 (9–229)	
ACD	24 (9–47)	0.9998	31 (12–54)	>0.9999
Citrate	162 (6–415)	0.9888	86 (20–274)	0.9999
Heparin	247 (39–415)	0.0032	1682 (284–3725)	0.0075
EDTA	194 (50–359)	0.0501	840 (86–1852)	0.4880
**2 h, RT**				
CTAD	55 (12–316)		196 (50–572)	
ACD	18 (3–77)	0.9930	79 (10–495)	0.9985
Citrate	214 (51–316)	0.4575	667 (33–2084)	0.9583
Heparin	119 (30–210)	0.5037	349 (14–3928)	0.4351
EDTA	386 (36–727)	<0.0001	2316 (400–8167)	0.0005
**6 h, 4°C**				
CTAD	24 (4–76)		42 (19–534)	
ACD	17 (11–24)	0.9984	48 (0–78)	0.9998
Citrate	49 (14–72)	0.0770	246 (28–424)	0.9986
Heparin	290 (159–583)	0.0002	3765 (1658–8143)	<0.0001
EDTA	283 (149–853)	<0.0001	1256 (107–2389)	0.2732
**6 h, RT**				
CTAD	69 (24–162)		197 (66–429)	
ACD	88 (64–241)	0.9930	163 (84–4540)	0.8107
Citrate	195 (102–355)	0.4575	417 (12–3194)	0.8107
Heparin	166 (61–384)	0.5037	2181 (12–3194)	0.0083
EDTA	467 (38–1492)	<0.0001	2163 (754–7143)	0.0008
**24 h, 4°C**				
CTAD	36 (9–83)		208 (46–346)	
ACD	65 (32–207)	0.8776	93 (2–186)	0.9990
Citrate	274 (49–558)	0.0019	502 (17–1016)	0.9703
Heparin	592 (102–838)	<0.0001	2925 (620–11402)	<0.0001
EDTA	595 (281–1165)	<0.0001	2752 (399–6954)	<0.0001
**24 h, RT**				
CTAD	225 (111–503)		602 (117–1061)	
ACD	199 (174–498)	0.9987	168 (12–1997)	0.9883
Citrate	362 (193–575)	0.6141	1927 (142–4870)	0.2144
Heparin	307 (105–609)	0.7573	1313 (185–5484)	0.1257
EDTA	1026 (217–1499)	<0.0001	5244 (2011–11360)	<0.0001

**Fig 4:**
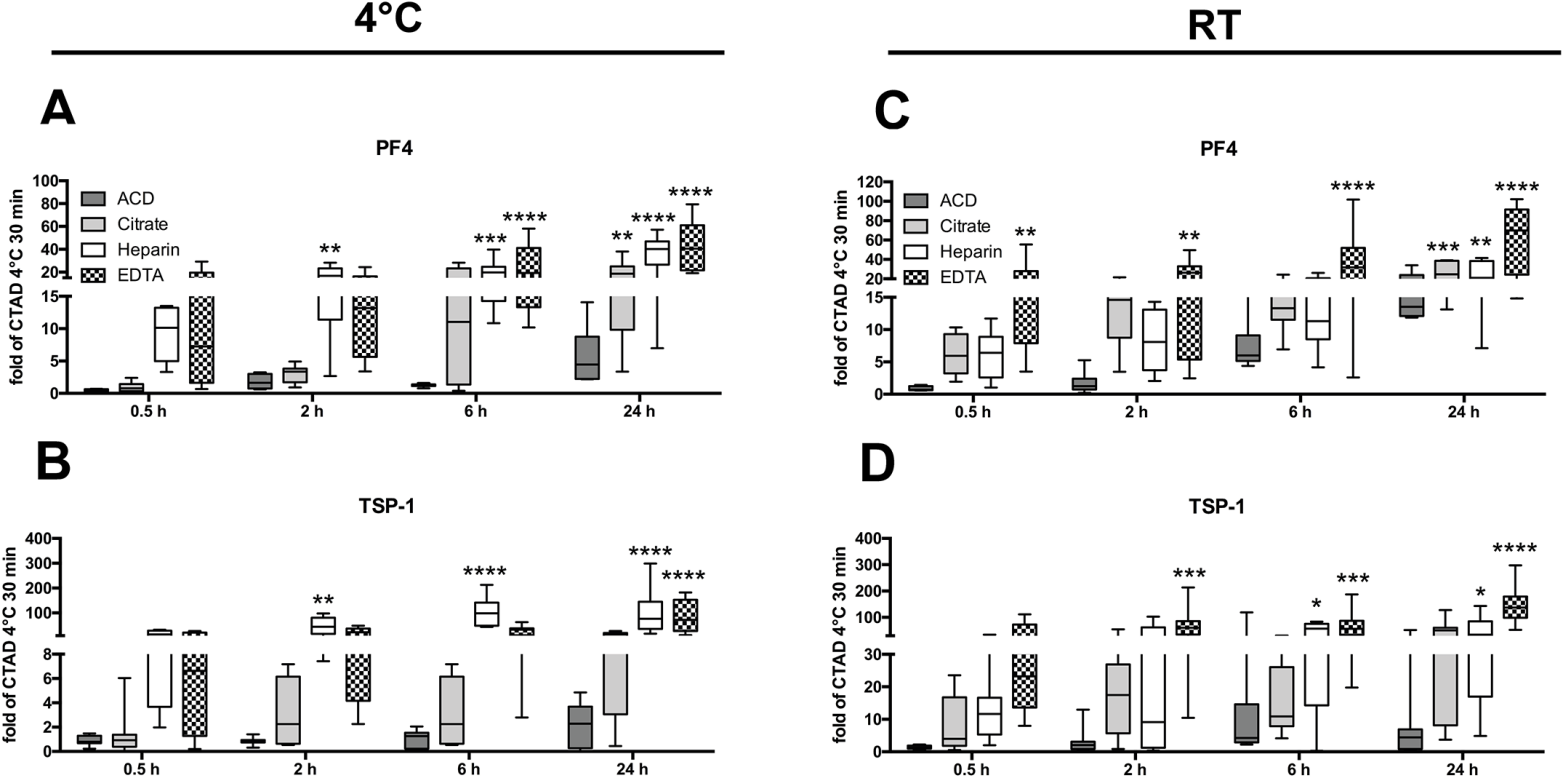
Fold-increase of platelet-stored factors due to suboptimal plasma generation. Plasma concentrations of PF4 (A, C) and TSP-1 (B, D) were calculated as fold of plasma levels relative to CTAD plasma 30 min 4°C (8 healthy donors). Fold-increase is depicted for ACD (dark grey bar), citrate (grey bar), heparin (white bar) or EDTA (patterned bar) plasma at 0.5 h, 2 h, 6 h, and 24 h. Samples were either processed at 4°C (A-B) or at ambient temperature (C-D). Significant differences were analyzed using one-way ANOVA with Dunnett correction and were depicted as *p<0.05, **p<0.01, ***p<0.001, and ****p<0.0001 (in comparison to CTAD 30 min 4°C).

For a quantification of the unwanted *in vitro* activation of platelets in comparison to plasma prepared with CTAD (30 min, 4°C), we calculated the fold-increase of the markers using the different other anticoagulants (Fig 4).

Anticoagulation with heparin and EDTA led to an increase in plasma levels of PF4 and TSP-1 even under conditions of immediate plasma preparation at 4°C (Fig 4A and 4B). Additionally, this effect increased even more after 2 hours and 6 hours in heparin and EDTA plasma. Citrate plasma showed comparable levels of PF4 and TSP-1 with CTAD plasma when processed at 4°C, however, after a time period of 24 hours the levels were increased 23-fold and 20-fold in comparison to optimized CTAD plasma preparation, respectively.

At room temperate, pronounced increases were already observed at 30 minutes with an 6-35-fold increase of PF4 and TSP-1 levels in citrate, heparin, and EDTA plasma (Fig 4C and 4D).

Plasma sCD62P levels were neither affected by anticoagulants, time or temperature (S2 Fig).

### Effect of *in vitro* platelet activation on release of platelet stored factors

**Fig 5:**
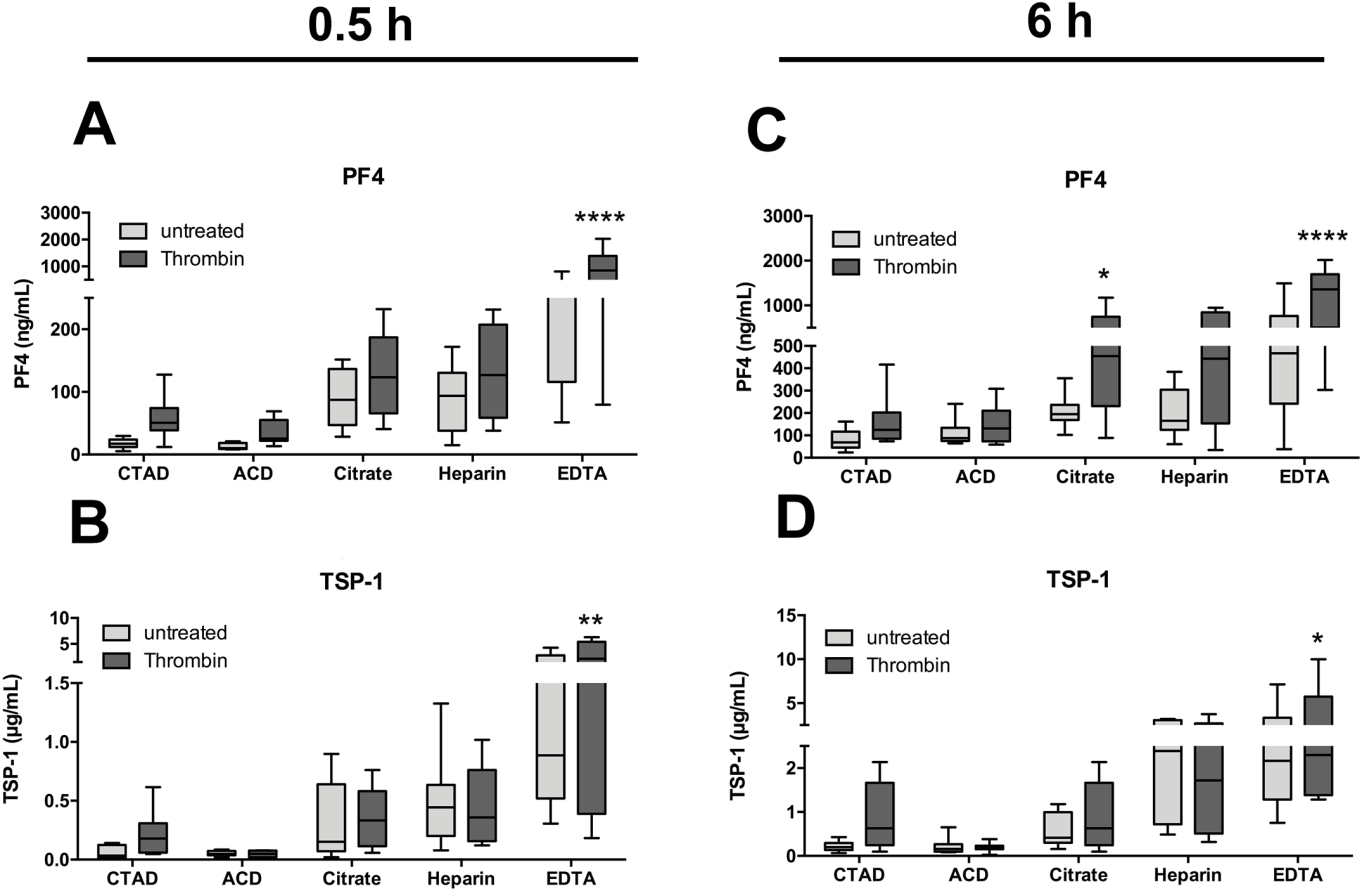
Effect of *in vitro* platelet activation on concentration of plasma factors. Blood of 8 healthy donors was anticoagulated with CTAD, ACD, citrate, heparin or EDTA (grey bar) and stimulated with the platelet activator thrombin (dark grey bar) at a submaximal dose for 0.5 h and 6 h at room temperature. Subsequently, plasma was prepared and analyzed for PF4 (A, C) and TSP-1 (B, D) levels. Significant differences were analyzed using two-way ANOVA with Bonferroni correction and were depicted as *p<0.05, **p<0.01, ***p<0.001, and ****p<0.0001 (in comparison to untreated).

Since suboptimal plasma preparation apparently results in artificial platelet activation, we aimed to test the effect of anticoagulants on provoked platelet activation (Fig 5). Stimulation of anticoagulated blood with submaximal concentrations of thrombin caused a mild increase in plasma PF4 and TSP-1 levels within all anticoagulants. However, PF4 levels of CTAD and ACD plasma, even when stimulated with thrombin, were lower than in unstimulated citrate, heparin and EDTA plasma. The most pronounced rise after thrombin treatment was observed in EDTA plasma with 849 ng/mL and 2140 ng/mL for PF4 and TSP-1, respectively (Fig 5A and 5B).

Increasing incubation time from 30 minutes to 6 hours only caused a minor increase in TSP-1 levels, but led to a marked rise in PF4 levels (Fig 5C and 5D). Levels of sCD62P were not affected by thrombin treatment (S3 Fig).

## Discussion

Our results clearly illustrate the importance of using CTAD as anticoagulant for the generation of platelet-free plasma before measuring plasma markers of *in vivo* platelet activation. This was not only depicted by its consistently low levels of PF4 and TSP-1 even after storage over 24 hours, but also by its uniquely low variation between different plasma preparations. Anticoagulation with citrate, heparin, and EDTA is commonly used to measure concentrations of plasma factors in clinical settings [14]. However, we can clearly show that these suboptimal plasma preparation methods lead to a substantial degree of *in vitro* platelet granule release, which makes measurements prone to *in vitro* artefacts. Therefore, we want to raise scientific awareness for choosing the optimal anticoagulant, which is not only important for studies primarily focusing on platelets and coagulation, but also applies to a much broader spectrum of research as platelets store a plethora of heterogeneous proteins, affecting inflammation, angiogenesis, wound healing, and coagulation.

The preservative effect of CTAD can be explained by its various compounds: citrate, theophylline, adenosine, and dipyridamole [15]. Platelet activation is curtailed by endogenous levels of cyclic adenosine 3’, 5’-monophosphate (cAMP) and cyclic guanosine-monophosphate (cGMP), which are kept high under physiological conditions due to release of nitric oxide and prostacyclin from the endothelium [16]. In blood processed in the presence of CTAD, this is achieved by adenosine, which stimulates adenylate cyclase, and by theophylline and dipyridamole, which inhibit phosphodiesterase-mediated cAMP degradation, thereby preventing *in vitro* activation of platelets [17–19]. Moreover, we could show that anticoagulation with ACD was similar effective as CTAD, which could be explained by the fact that ACD lowers the pH of blood to 6.5 thereby preventing platelet activation and aggregation [20].

Both citrate and EDTA chelate calcium, which is required for platelet activation and secondary hemostasis. A previous study suggested that EDTA may cross platelet plasma membranes to chelate intracellular calcium to prevent platelet activation [21]. However, our results demonstrate that *in vitro* platelet activation does occur in EDTA-anticoagulated blood as plasma levels of PF4 and TSP-1 were highest in EDTA plasma at 4°C and at room temperature. Of note, we could observe hemolysis in most of the plasma samples generated from EDTA blood, potentially affecting the quantification of certain plasma factors by colorimetric assays.

All three measured platelet factors, PF4, TSP-1, and sCD62P, are stored within α-granules, which is the largest and most abundant platelet granule type. The content of α-granules either arises from packaging within megakaryocytes or *via* uptake from the plasma by endocytosis, which makes platelets environmental sensors to changes in extracellular milieu [22] and an important surrogate marker for various disease. However, we could clearly demonstrate that α-granules are also prone to be released artificially due to suboptimal plasma preparation with EDTA or heparin blood, strengthening the importance of using proper anticoagulants, which not only prevents clotting, but also suppresses platelet activation and subsequent granule release. It is important to note that the standard deviation of biomarkers substantially increased in these suboptimal plasma preparations, suggesting that, even if all samples within a clinical trial were collected with the same sample preparation method, the degree of *in vitro* platelet activation during processing substantially varies between samples.

Moreover, our results clearly point out that storage temperature as well as time are additional factors affecting platelet activation, which has to be carefully considered during clinical monitoring of plasma concentrations of platelet granule contents. We provide evidence that blood anticoagulated with CTAD or ACD was able to provide the most robust and reproducible values, even when processed at ambient temperature or after storage for 6 hours, whereas anticoagulants such as heparin and EDTA led to a significant release of platelet granules. This is also reflected in studies showing that CTAD is preferable as an anticoagulant for flow cytometric measurement of platelet activation [23–25].

Furthermore, we did not only study the effect of time and temperature, but additionally challenged different anticoagulants by artificially activating platelets *in vitro* using a submaximal dose of the potent platelet activator thrombin. Thrombin is generated in small amounts e.g. by suboptimal blood drawing and therefore may lead to platelet activation *via* protease-activated receptor-1 (PAR-1) and PAR-4 [26]. Although thrombin caused a moderate rise of PF4 and TSP-1 levels in all plasma samples, levels of CTAD and ACD plasma still remained markedly low, underlining its robustness to artificial platelet activation during processing and therefore the importance of its usage in clinical settings.

In contrast to PF4 and TSP-1, we observed only moderate changes in sCD62P plasma content due to anticoagulants, time or temperature, with low sCD62P levels in all samples. CD62P is a transmembrane protein and as such a component of the α-granule membranes, which is exposed on the platelet surface upon activation. For release into the soluble phase of the plasma it requires cleavage and shedding from the membranes [27, 28], which might not occur under the conditions of plasma preparation that we used, explaining that hardly any changes could be detected. Therefore, sCD62P proved to be insufficient as plasma marker of *in vitro* platelet activation as α-granule release and proteolysis of CD62P from the surface of platelets clearly represent two distinct events.

Taken together, our data strongly suggest the use of on optimized plasma preparation using CTAD or ACD anticoagulated blood for the detection of platelet-derived plasma factors, since suboptimal plasma preparation leads to artificial *in vitro* platelet activation, which might ultimately mask biologically relevant differences within translational research studies.

## Supporting information

S1 Fig Plasma levels of sCD62P do not vary among different anticoagulants. Plasma levels of CD62P (A, B) were determined in 8 healthy individuals. Plasma was prepared from CTAD (black bar), citrate (dark grey bar), heparin (grey bar) or EDTA (white bar) blood at 4°C (A) or at room temperature (B) within 30 min after blood draw. Plasma concentration was measured using ELISA. Significant differences were analyzed using one-way ANOVA with Dunnett correction.
(TIF)

S2 Fig Time-dependent differences in plasma levels of sCD62P. Blood was anticoagulated with CTAD (black bar), citrate (dark grey bar), heparin (grey bar) or EDTA (white bar) and stored at 4°C (A-B) or at room temperature (C-D) for 0.5 h, 2 h, 6 h or 24 h until plasma preparation. Total concentrations of sCD62P (A, C) and fold concentrations in comparison to CTAD 4°C 30 min were determined for each time point. Significant differences were analyzed using two-way ANOVA with Dunnett correction (with CTAD as reference).
(TIF)

S3 Fig Effect of *in vitro* platelet activation on concentration of sCD62P. Blood was anticoagulated with CTAD, citrate, heparin or EDTA (grey bar) and stimulated with the platelet activator thrombin (dark grey bar) at a submaximal dose for 0.5 h and 6 h at room temperature. Subsequently, plasma was prepared and analyzed for sCD62P (A, B) levels. Significant differences were analyzed using two-way ANOVA with Bonferroni correction.
(TIF)

## References

[1] Tuck MK, Chan DW, Chia D, Godwin AK, Grizzle WE, Krueger KE, et al Standard operating procedures for serum and plasma collection: early detection research network consensus statement standard operating procedure integration working group. J Proteome Res. 2009;8(1):113–7. doi: 10.1021/pr800545q 19072545 10.1021/pr800545q PMC2655764

[2] Ammerlaan W, Trezzi JP, Lescuyer P, Mathay C, Hiller K, Betsou F. Method validation for preparing serum and plasma samples from human blood for downstream proteomic, metabolomic, and circulating nucleic acid-based applications. Biopreserv Biobank. 2014;12(4):269–80. Epub 2014/07/30. doi: 10.1089/bio.2014.0003 . 25075813 10.1089/bio.2014.0003

[3] Mohamadkhani A, Poustchi H. Repository of Human Blood Derivative Biospecimens in Biobank: Technical Implications. Middle East J Dig Dis. 2015;7(2):61–8. 26106464 PMC4430793

[4] Battinelli EM, Markens BA, Italiano JE. Release of angiogenesis regulatory proteins from platelet alpha granules: modulation of physiologic and pathologic angiogenesis. Blood. 2011;118(5):1359–69. Epub 2011/06/16. doi: 10.1182/blood-2011-02-334524 21680800 10.1182/blood-2011-02-334524 PMC3152500

[5] Golebiewska EM, Poole AW. Platelet secretion: From haemostasis to wound healing and beyond. Blood Rev. 2015;29(3):153–62. Epub 2014/10/31. doi: 10.1016/j.blre.2014.10.003 25468720 10.1016/j.blre.2014.10.003 PMC4452143

[6] Kral JB, Schrottmaier WC, Salzmann M, Assinger A. Platelet Interaction with Innate Immune Cells. Transfus Med Hemother. 2016;43(2):78–88. Epub 2016/03/09. doi: 10.1159/000444807 27226790 10.1159/000444807 PMC4872052

[7] Assinger A. Platelets and infection—an emerging role of platelets in viral infection. Front Immunol. 2014;5:649 Epub 2014/12/18. doi: 10.3389/fimmu.2014.00649 25566260 10.3389/fimmu.2014.00649 PMC4270245

[8] Martinez MC, Andriantsitohaina R. Microparticles in angiogenesis: therapeutic potential. Circ Res. 2011;109(1):110–9. doi: 10.1161/CIRCRESAHA.110.233049 . 21700952 10.1161/CIRCRESAHA.110.233049

[9] Rendu F, Brohard-Bohn B. The platelet release reaction: granules' constituents, secretion and functions. Platelets. 2001;12(5):261–73. doi: 10.1080/09537100120068170 . 11487378 10.1080/09537100120068170

[10] Senzel L, Gnatenko DV, Bahou WF. The platelet proteome. Curr Opin Hematol. 2009;16(5):329–33. doi: 10.1097/MOH.0b013e32832e9dc6 19550320 10.1097/MOH.0b013e32832e9dc6 PMC2883290

[11] Jonnalagadda D, Izu LT, Whiteheart SW. Platelet secretion is kinetically heterogeneous in an agonist-responsive manner. Blood. 2012;120(26):5209–16. Epub 2012/10/18. doi: 10.1182/blood-2012-07-445080 23086755 10.1182/blood-2012-07-445080 PMC3537312

[12] Starlinger P, Haegele S, Offensperger F, Oehlberger L, Pereyra D, Kral JB, et al The profile of platelet α-granule released molecules affects postoperative liver regeneration. Hepatology. 2016;63(5):1675–88. Epub 2015/12/28. doi: 10.1002/hep.28331 . 26528955 10.1002/hep.28331

[13] Starlinger P, Alidzanovic L, Schauer D, Brugger P, Sommerfeldt S, Kuehrer I, et al Platelet-stored angiogenesis factors: clinical monitoring is prone to artifacts. Dis Markers. 2011;31(2):55–65. doi: 10.3233/DMA-2011-0798 21896999 10.3233/DMA-2011-0798 PMC3826483

[14] Bowen RA, Remaley AT. Interferences from blood collection tube components on clinical chemistry assays. Biochem Med (Zagreb). 2014;24(1):31–44. Epub 2014/02/15. doi: 10.11613/BM.2014.006 24627713 10.11613/BM.2014.006 PMC3936985

[15] Yokota M, Tatsumi N, Tsuda I, Nishioka T, Takubo T. CTAD as a universal anticoagulant. J Autom Methods Manag Chem. 2003;25(1):17–20. doi: 10.1155/S1463924603000038 18924886 10.1155/S1463924603000038 PMC2548379

[16] Smolenski A. Novel roles of cAMP/cGMP-dependent signaling in platelets. J Thromb Haemost. 2012;10(2):167–76. doi: 10.1111/j.1538-7836.2011.04576.x . 22136590 10.1111/j.1538-7836.2011.04576.x

[17] Salzman EW, Kensler PC, Levine L. Cyclic 3',5'-adenosine monophosphate in human blood platelets. IV. Regulatory role of cyclic amp in platelet function. Ann N Y Acad Sci. 1972;201:61–71. . 4346063 10.1111/j.1749-6632.1972.tb16287.x

[18] Mills DC, Smith JB. The influence on platelet aggregation of drugs that affect the accumulation of adenosine 3':5'-cyclic monophosphate in platelets. Biochem J. 1971;121(2):185–96. 4330088 10.1042/bj1210185 PMC1176554

[19] Paul S, Feoktistov I, Hollister AS, Robertson D, Biaggioni I. Adenosine inhibits the rise in intracellular calcium and platelet aggregation produced by thrombin: evidence that both effects are coupled to adenylate cyclase. Mol Pharmacol. 1990;37(6):870–5. . 2359405

[20] ASTER RH, JANDL JH. PLATELET SEQUESTRATION IN MAN. I. METHODS. J Clin Invest. 1964;43:843–55. doi: 10.1172/JCI104970 14169513 10.1172/JCI104970 PMC289563

[21] Ogundele MO. Bimodal role for divalent cations in the mechanism of EDTA cytolysis. Br J Biomed Sci. 2000;57(4):312–5. . 11204862

[22] Blair P, Flaumenhaft R. Platelet alpha-granules: basic biology and clinical correlates. Blood Rev. 2009;23(4):177–89. Epub 2009/05/17. doi: 10.1016/j.blre.2009.04.001 19450911 10.1016/j.blre.2009.04.001 PMC2720568

[23] Mody M, Lazarus AH, Semple JW, Freedman J. Preanalytical requirements for flow cytometric evaluation of platelet activation: choice of anticoagulant. Transfus Med. 1999;9(2):147–54. . 10354385 10.1046/j.1365-3148.1999.00188.x

[24] Neufeld M, Nowak-Göttl U, Junker R. Citrate-theophylline-adenine-dipyridamol buffer is preferable to citrate buffer as an anticoagulant for flow cytometric measurement of platelet activation. Clin Chem. 1999;45(11):2030–3. . 10545083

[25] Kühne T, Hornstein A, Semple J, Chang W, Blanchette V, Freedman J. Flow cytometric evaluation of platelet activation in blood collected into EDTA vs. Diatube-H, a sodium citrate solution supplemented with theophylline, adenosine, and dipyridamole. Am J Hematol. 1995;50(1):40–5. . 7668222 10.1002/ajh.2830500108

[26] Kahn ML, Nakanishi-Matsui M, Shapiro MJ, Ishihara H, Coughlin SR. Protease-activated receptors 1 and 4 mediate activation of human platelets by thrombin. J Clin Invest. 1999;103(6):879–87. doi: 10.1172/JCI6042 10079109 10.1172/JCI6042 PMC408153

[27] Berger G, Hartwell DW, Wagner DD. P-Selectin and platelet clearance. Blood. 1998;92(11):4446–52. . 9834252

[28] Michelson AD, Barnard MR, Hechtman HB, MacGregor H, Connolly RJ, Loscalzo J, et al In vivo tracking of platelets: circulating degranulated platelets rapidly lose surface P-selectin but continue to circulate and function. Proc Natl Acad Sci U S A. 1996;93(21):11877–82. 8876231 10.1073/pnas.93.21.11877 PMC38152

