# Supplementary figures and images for: Optimized plasma preparation is essential to monitor platelet-stored molecules in humans
Source: PLoS One. 2017 Dec 8;12(12):e0188921. doi: 10.1371/journal.pone.0188921 (PMC5722331; doi:10.1371/journal.pone.0188921)

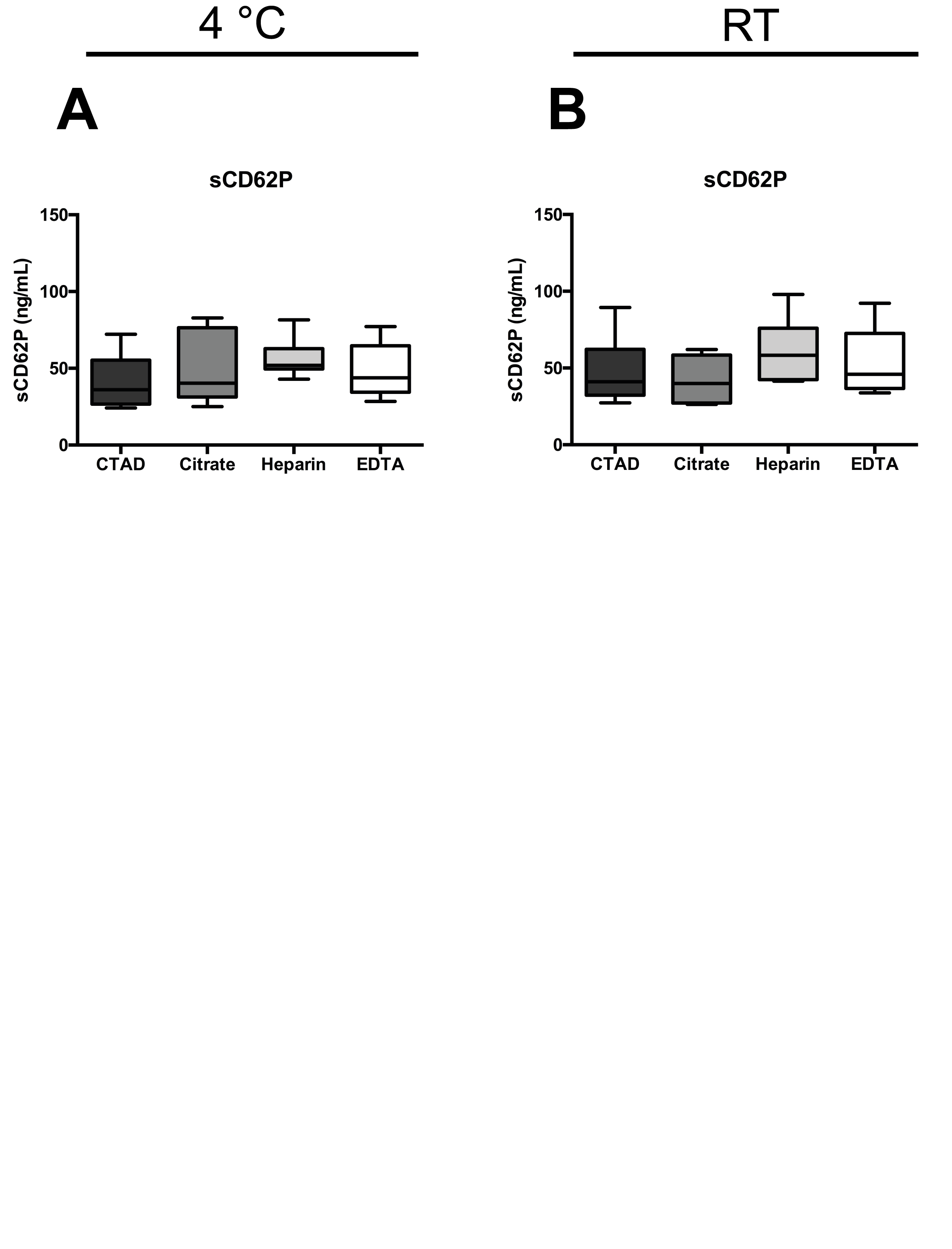

Supplement: S1 Fig — Plasma levels of CD62P (A, B) were determined in 8 healthy individuals. Plasma was prepared from CTAD (black bar), citrate (dark grey bar), heparin (grey bar) or EDTA (white bar) blood at 4°C (A) or at room temperature (B) within 30 min after blood draw. Plasma concentration was measured using ELISA. Significant differences were analyzed using one-way ANOVA with Dunnett correction. (TIF) [file pone.0188921.s001.tif]

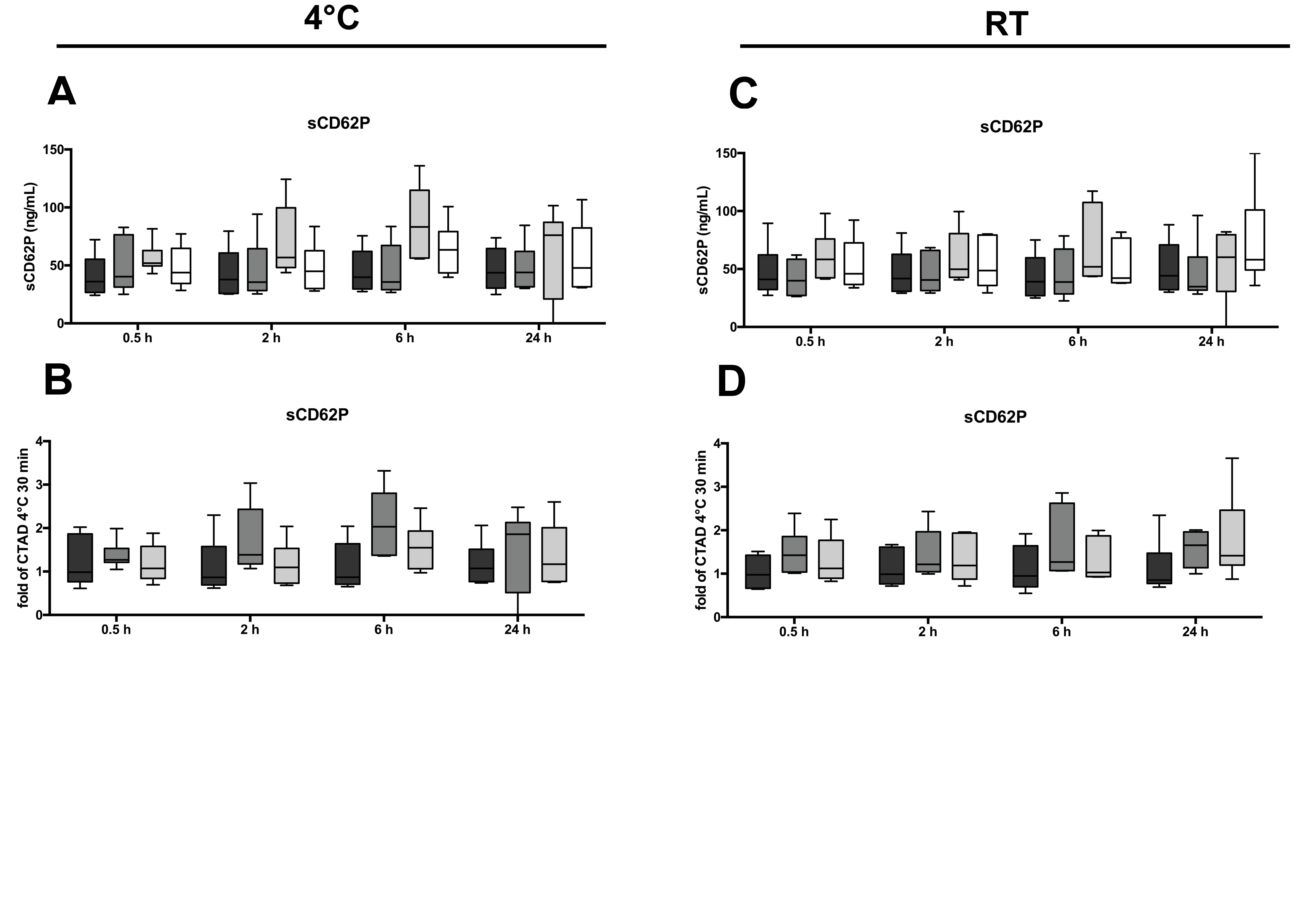

Supplement: S2 Fig — Blood was anticoagulated with CTAD (black bar), citrate (dark grey bar), heparin (grey bar) or EDTA (white bar) and stored at 4°C (A-B) or at room temperature (C-D) for 0.5 h, 2 h, 6 h or 24 h until plasma preparation. Total concentrations of sCD62P (A, C) and fold concentrations in comparison to CTAD 4°C 30 min were determined for each time point. Significant differences were analyzed using two-way ANOVA with Dunnett correction (with CTAD as reference). (TIF) [file pone.0188921.s002.tif]

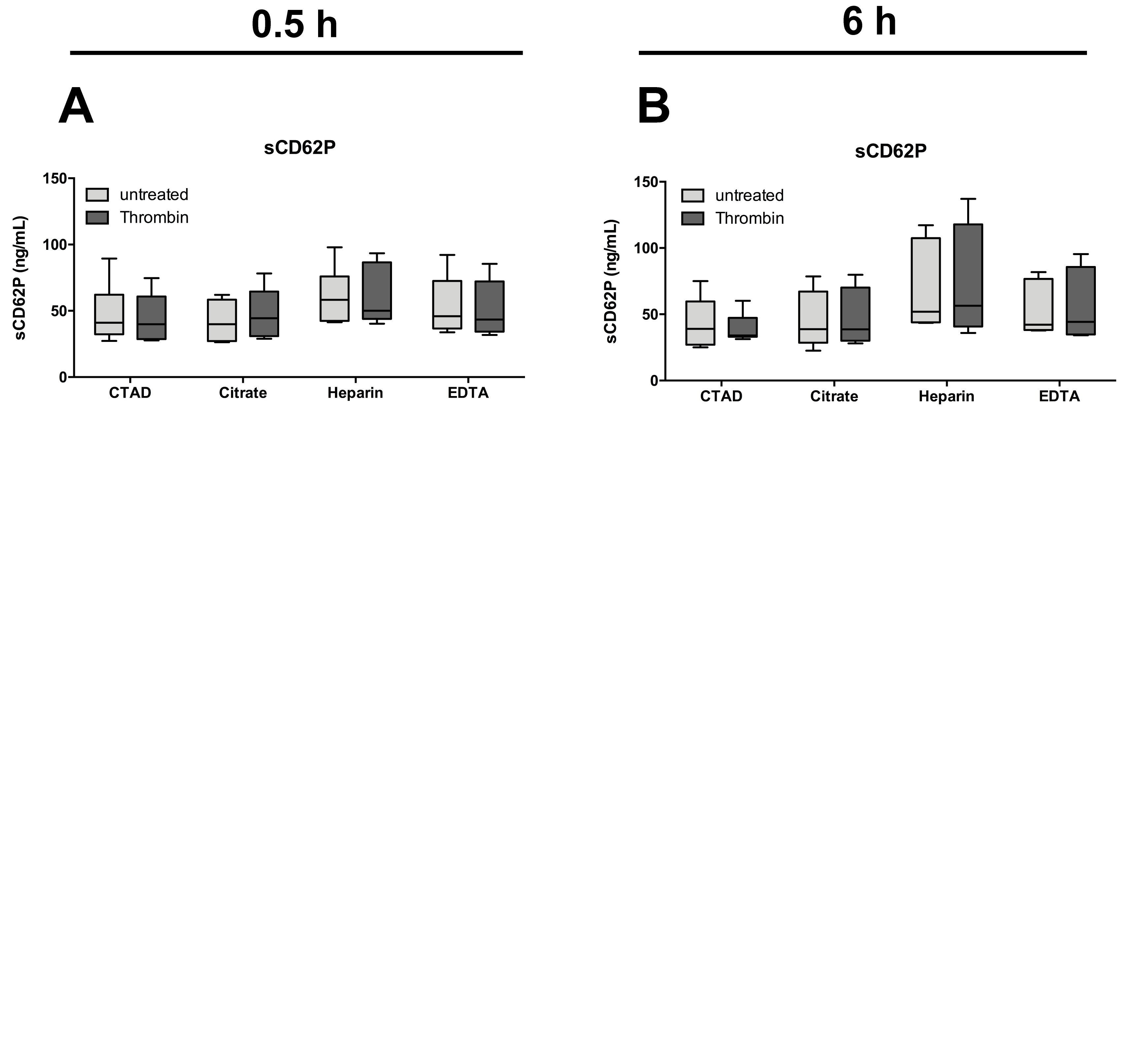

Supplement: S3 Fig — Blood was anticoagulated with CTAD, citrate, heparin or EDTA (grey bar) and stimulated with the platelet activator thrombin (dark grey bar) at a submaximal dose for 0.5 h and 6 h at room temperature. Subsequently, plasma was prepared and analyzed for sCD62P (A, B) levels. Significant differences were analyzed using two-way ANOVA with Bonferroni correction. (TIF) [file pone.0188921.s003.tif]
